# First record of *Ixodes luciae* (Acari: Ixodidae) parasitizing *Marmosops noctivagus* (Didelphimorphia: Didelphidae) in South America

**DOI:** 10.1590/S1984-29612025039

**Published:** 2025-08-07

**Authors:** Marcelo Cutrim Moreira de Castro, José Albertino Rafael, Fernando de Castro Jacinavicius, Darci Moraes Barros-Battesti, Ricardo Bassini-Silva

**Affiliations:** 1 Secretaria Municipal de Educação – SEMED, Divisão de Desenvolvimento Profissional do Magistério, Manaus, AM, Brasil; 2 Instituto Nacional de Pesquisas da Amazônia – INPA, Programa de Pós-graduação em Entomologia, Manaus, AM, Brasil; 3 Universidade Estadual de Campinas – UNICAMP, Instituto de Biologia, Departamento de Biologia Animal, Campinas, SP, Brasil; 4 Universidade Estadual Paulista – UNESP, Faculdade de Ciências Agrárias e Veterinárias – FCAV, Departamento de Patologia, Reprodução e Saúde Única, Jaboticabal, SP, Brasil; 5 Instituto Butantan, Laboratório de Coleções Zoológicas, São Paulo, SP, Brasil

**Keywords:** Ectoparasites, marsupials, parasitism, Amazon, host-parasite interaction, Ectoparasitos, marsupiais, parasitismo, Amazônia, interação parasito-hospedeiro

## Abstract

This study reports the first record of parasitism of *Ixodes luciae* Sénevet, on the marsupial *Marmosops noctivagus* (Tschudi), in Brazil and in South America. The tick *I. luciae*, previously recorded in Brazil in the Amazonas state, was collected from the marsupial using a flight intercept trap suspended 28 meters above ground, at the canopy level, in an area located 60 km north from Manaus, Amazonas, Brazil. It is the first time this marsupial has been collected with this type of trap, representing a bycatch collection. Both records highlight significant contributions to better understanding host-parasite interactions and the biodiversity of the Amazonian ecosystem. Both 18S rRNA and 16S rRNA sequences showed 100% identity with previously available *I. luciae* sequences. Molecular analysis did not indicate the presence of Rickettsiales agents in *I. luciae*.

## Resumo

Este estudo relata o primeiro registro de parasitismo de *Ixodes luciae* Sénevet, no marsupial *Marmosops noctivagus* (Tschudi), representando o primeiro registro dessa associação no Brasil e na América do Sul. O carrapato *I. luciae*, previamente registrado no Brasil, no estado do Amazonas, foi coletado do marsupial capturado em uma armadilha de interceptação de vôo para insetos suspensa a 28 metros do solo, ao nível da copa das árvores, em uma área localizada a 60 km ao norte de Manaus, Amazonas, Brasil. É também a primeira vez que este marsupial foi coletado com este tipo de armadilha, representando uma coleta acidental. Ambos registros ressaltam contribuições significativas para uma melhor compreensão das interações hospedeiro-parasito e da biodiversidade do ecossistema amazônico. Tanto a sequência de 18S rRNA quanto a de 16S rRNA mostraram 100% de identidade com as sequências prévias disponíveis de *I. luciae*. A análise molecular não indicou a presença de DNA de agentes Rickettsiales em *I. luciae*.

The Ixodidae Koch, 1844 ticks are known as hard ticks due to the presence of a large antero-dorsal sclerite, or shield, in all life stages. They exhibit marked sexual dimorphism, such that males have a posterior edge of the opisthosoma that may display sclerotized dorsal structures called festoons (as seen in *Amblyomma* species) or ventral plates (as in *Rhipicephalus* species), which are absent in females (Barros-Battesti et al., 2024).

In Brazil, this family is represented by 53 species, of which 34 belong to *Amblyomma* Koch, 1844, 12 to *Ixodes* Latreille, 1795 and seven to other genera: *Dermacentor* Koch, 1844, *Haemaphysalis* Koch, 1844 and *Rhipicephalus* Koch, 1844. All of these are considered important due to blood-feeding and pathogen transmission (Barros-Battesti et al., 2024). In the Brazilian Amazon region, 37 species occur, with 30 already reported in the Amazonas (Barros-Battesti et al., 2024) state. In this federal state, only a few studies on ectoparasites have been conducted in the municipality of Manaus (Gianizella et al., 2018).

*Ixodes* consists of 283 described species worldwide, including two fossil species. Of these, 62 occur in the Neotropical region, 12 in Brazil, four in the Brazilian Amazon region, and two in the Amazonas state: *Ixodes bocatorensis* Apanaskevich and Bermúdez, 2017, and *Ixodes luciae* Sénevet, 1940 (Barros-Battesti et al., 2024).

*Ixodes luciae* has been reported from Argentina, Belize, Bolivia, Brazil, Colombia, Costa Rica, Ecuador, Guatemala, French Guiana, Honduras, Mexico, Nicaragua, Panama, Peru, Suriname, Trinidad & Tobago and Venezuela (Díaz et al., 2009; Martins et al., 2024). In Brazil, it occurs in the North region, including Amazonas state (Gianizella et al., 2018), Acre, Pará and Rondônia (Martins et al., 2024) states, as well as in the Northeast region (Maranhão state) (Costa et al., 2020) and Central-Western region (Mato Grosso and Mato Grosso do Sul states) (Labruna et al., 2005; Witter et al., 2016; Martins et al., 2024). Its preferred hosts are marsupials during adult stages and small mammals, mainly rodents, during juvenile stages (Guimarães et al., 2001). However, there are records of marsupials also infested with larvae and nymphs (Autino et al., 2006).

The white-bellied slender mouse opossum, *Marmosops noctivagus* (Tschudi 1845), is a small mammal in the Didelphidae family (Didelphimorphia). In Brazil, it is found in the northwestern region, in Acre, Amazonas, Mato Grosso and Pará (Gardner, 2005) states. It inhabits mainly primary, secondary and disturbed forests, being nocturnal, insectivorous-omnivorous, solitary and arborial. It uses both the ground and lower vegetation strata to actively move through fallen trees, dense understory clumps and vines (Emmons & Feer, 1997; Vieira & Camargo, 2012).

One of the methods for capturing insects in the upper stratum of the forest canopy is the suspended Malaise trap. This trap creates a microenvironment teeming with insects that can ferment alcohol, making it an attractive food source for predators. Many of these predators are small Didelphidae, which have a varied diet that includes small vertebrates, fruits, leaves, seeds, nectar, plant exudates, fungi and insects (Amador & Giannini, 2020). This study describes the intriguing record of a tick on a marsupial (*Marmosops noctivagus*) captured in an insect trap set in the forest canopy.

**Figure 1:**
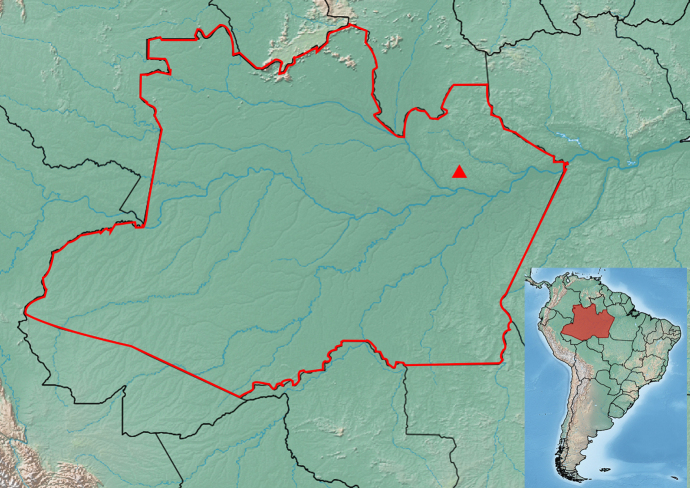
Location of the host and ectoparasite collecting site in Manaus municipality, Amazonas State, northern Brazil.

The marsupial *M. noctivagus*, carrying the tick, was collected in June 2024 using a flight interception trap for insects, placed at a height of 28 m in the forest canopy. The trap was installed in an Amazonian biogeographical component at the Tropical Silviculture Experimental Station which belongs to the National Institute of Amazonian Research (INPA), located on the ZF-2 road (02°35′33′S and 60°06′55′W), 60 km north of Manaus, Amazonas (Figure 1). The specimen was placed in a container with 96% ethyl alcohol and was transported to and analyzed at the Urban and Forensic Systematic Entomology Laboratory (INPA). Its body mass (BM) in grams, length of tail (LT), total length (nose to tail-tip, TL) and head-and-body length (HBL) in millimeters were measured. Its entire external surface, including the fur, was inspected for tick collection, which was performed using entomological tweezers.

The morphological identification of the tick was performed following specific taxonomic literature (Nava et al., 2017; Onofrio et al., 2010). The main morphological characteristics identified in the studied *I. luciae* nymph specimen are the basis capituli long, triangular; cornua absent; venter of basis with large lateral extensions homologous to auriculae; scutum elongate with long, distinct lateral carinae, scapulae short and rounded; and mainly external spur on coxa I longer and larger than internal; hypostome pointed.

The voucher material was deposited in the Acarological Collection of the Zoological Collections, Butantan Institute (IBSP). Photographs of the marsupial and tick specimens were taken using a Leica DFC500 digital camera coupled to a Leica M205c microscope, connected to a computer using the Leica Application Suite LAS V3.6 software, which includes an automontage module.

For molecular identification, the entire specimen DNA material was placed in a 1.5 µL Eppendorf® microtube and was extracted using the QIAGEN DNeasy Blood & Tissue kit, following the manufacturer’s protocols. Two molecular markers (18S rRNA and 16S rRNA) were targeted for molecular characterization of this tick species. The following primers and their respectively PCR protocols were used as described in the original articles to target each gene fragment: 18S rRNA using the primers Mite18S-1F (5′-ATATTGGAGGGCAAGTCTGG-3′) and Mite18S-1R (5′-TGGCATCGTTTATGGTTAG-3′); and 16S rRNA using the primers 16S+1 (5′-CTGCTCAATGATTTTTTAAATTGCTGTGG-3′) and 16S–1(5′-CCGGTCTGAACTCAGATCAAGT-3′) (Mangold et al., 1998). The PCR reagent concentration and thermal cycler conditions were as described in the abovementioned studies. A negative control (ultrapure water type I) and a positive control (pool of *Tyrophagus* sp.) were used for each reaction.

After extraction, the DNA was subjected to additional convective PCRs (cPCRs) in order to detect Anaplasmataceae agents and *Rickettsia* spp. For this purpose, PCRs targeting *16S rRNA* and *gltA*, respectively, were performed. A negative control (Milli-Q DNA-free water) and a positive control were included in each reaction (Labruna et al., 2004).

The PCR product was purified using ExoSap-IT (GE Healthcare, Pittsburgh, PA, USA). Sanger sequencing was performed at the Human Genome and Stem Cell Research Center of the Biosciences Institute, University of São Paulo, São Paulo, SP, Brazil. The sequence thus obtained was assembled using the Sequencing Analysis 5.3.1 software and was subjected to BLAST analysis (Altschul et al., 1990) to infer similarities with other sequences available in GenBank.

**Figure 2:**
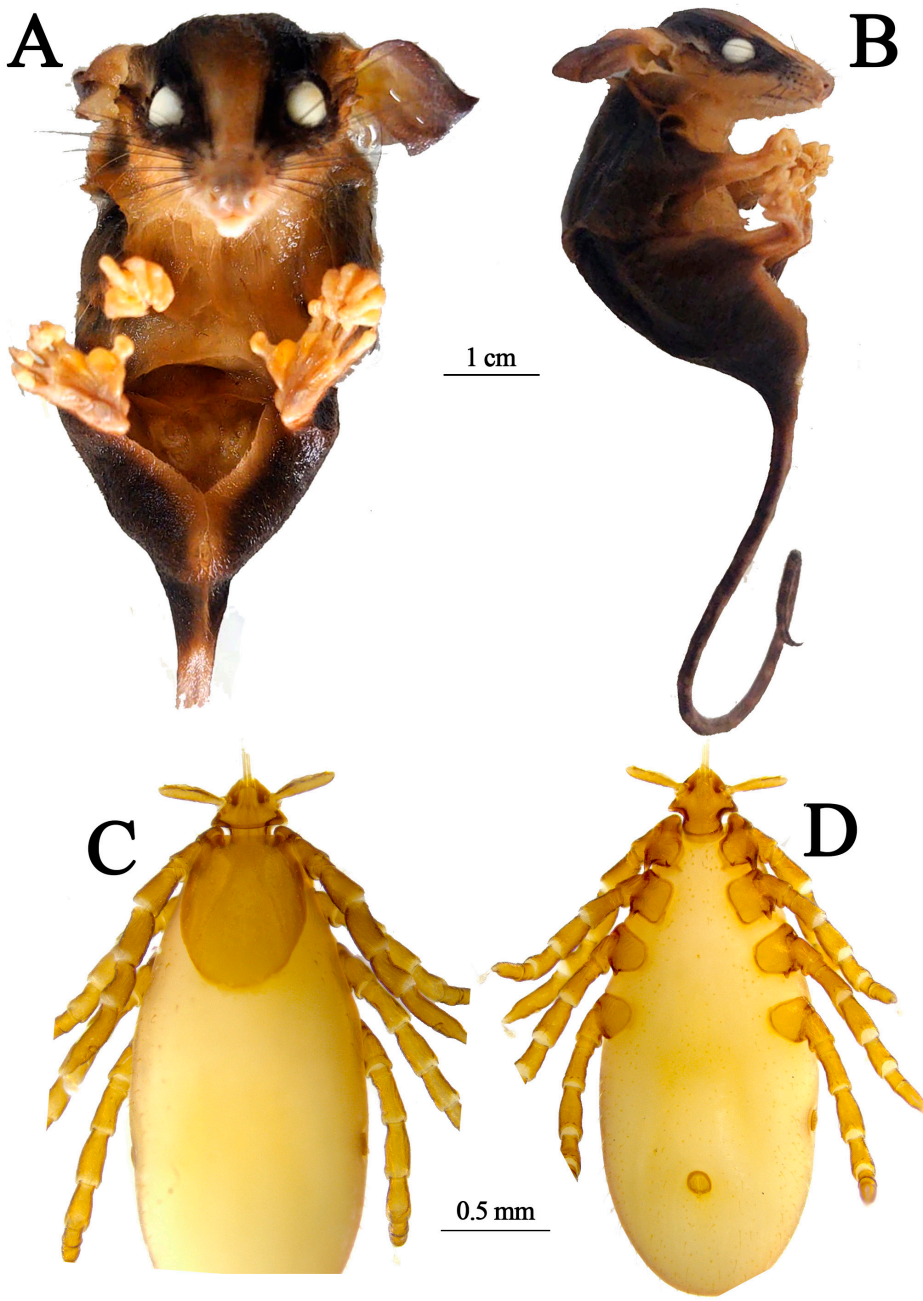
*Marmosops noctivagus* subadult, (A) Ventral view; (B) Lateral view (scales in cm); *Ixodes luciae* nymph; (C) Dorsal view; (D) Ventral view (scales in mm).

The *M. noctivagus* (Figure 2A and 2B) specimen possesses the following measurements: BM = 14, TL = 185, LT = 105 and HBL = 85. These values were lower than those reported by Voss et al. (2004), suggesting that the specimen was not an adult. This species has previously been recorded in the Amazon, specifically in the Amazonas (Emmons & Feer, 1997; Vieira & Camargo, 2012) state. In the present report, the specimen was collected in the tree canopy indicating that although *M. noctivagus* primarily inhabits the understory, this species may expand its vertical exploration range into higher strata when searching for resources such as food. The closely related species of the Atlantic Forest, *Marmosops incanus*, has also been recorded in upper strata but does not reach the canopy (Cunha & Vieira, 2002).

A single nymph of the tick *I. luciae* (Figure 2C and 2D) was found in the dorsal region of the host's neck. In addition to the morphological identification, DNA was successfully extracted from the specimen, and a partial sequence of the genes tested (18S rRNA and 16S rRNA) was obtained. The GenBank accession numbers for these sequences are PV113350 and PV113434, respectively.

Upon comparison with sequences available in the GenBank database, the identification of *I. luciae* was corroborated. Our 18S rRNA and 16S rRNA sequences showed 100% shared identity (query cover: 100%; e-value: 0) with the *I. luciae* sequence (GenBank accession number: AF115367 and U95894, respectively).

Regarding parasitism, in Brazil, there are records of *I. luciae* parasitizing other marsupials, such as *Philander opossum* in Mato Grosso (Witter et al., 2016), and Mato Grosso do Sul (host not identified) (Onofrio et al., 2010) states in the Central-West region. In the Northeast region, it has been recorded parasitizing *Didelphis marsupialis* in Maranhão (Costa et al., 2020) state. In the North region, both nymphs and adults have been found in marsupials in Pará (Luz et al., 2013), Rondônia (Labruna et al., 2005) and Acre (*D. marsupialis*) (Tojal et al., 2021) states. In the Amazonas state, the first record of this tick was obtained from *D. marsupialis* (Onofrio, 2003) in the municipality of Santa Isabel do Rio Negro, and the second from *Micoureus demerarae* (Gianizella et al., 2018) in the municipality of Manaus. Therefore, the record in the present study represents the second occurrence of this tick in the city of Manaus, the third to the Amazonas state and the first documented case of *I. luciae* parasitizing *M. noctivagus* in Brazil and South America.

There is a significant overlap of the distribution areas of this tick and its host. The distribution of the marsupial (Acre, Amazonas, Pará and Mato Grosso) nearly coincides with the distribution of the tick (Acre, Amazonas, Pará, Rondônia, Maranhão, Mato Grosso and Mato Grosso do Sul), and encompasses both the Brazilian and the Peruvian Amazon regions.

No pathogens were detected in the *I. luciae* tick collected in the present study. According to Binetruy et al. (2020), the bacterium *Rickettsia bellii* was identified in *I. luciae* in French Guiana, a bacterium that is not pathogenic to humans. Additionally, there is a record of a female of this tick parasitizing a human in Argentina (Ivancovich & Luciani, 1992). In Brazil, this tick was tested for the presence of *R. bellii* in Mato Grosso state, with negative results (Witter et al., 2016). These findings corroborate the results obtained by Díaz et al. (2009), who also found no evidence that *I. luciae* serves as a possible vector of pathogens to humans.

Understanding the distribution of this species, both in horizontal and vertical strata, is crucial for comprehending ecological mechanisms and for developing more targeted and effective conservation strategies. These new records enhance our understanding of the natural cycle of this tick species and the ecological relationships between this parasite and its host, such as their coexistence within the same area.

## Data Availability

Data will be made available on request.
